# Integrative analysis of Mendelian randomization and gene expression profiles reveals a null causal relationship between adiponectin and diabetic retinopathy

**DOI:** 10.1080/21623945.2023.2234522

**Published:** 2023-07-17

**Authors:** Ao Zhang, Hui Wu, Chi Wang, Suyan Tian

**Affiliations:** a Department of Neurology, The First Hospital of Jilin University, Changchun, Jilin, P.R. China; b Department of Ophthalmology, The First Hospital of Jilin University, Changchun, Jilin, P.R. China; c Department of Internal Medicine, College of Medicine, and Markey Cancer Center, University of Kentucky, Lexington, KY, USA; d Division of Clinical Research, The First Hospital of Jilin University, Changchun, Jilin, P.R. China

**Keywords:** Mendelian randomization, gene expression profiles, adiponectin, diabetic retinopathy

## Abstract

Observational studies have been conducted to investigate the correlation between adiponectin and diabetic retinopathy (DR), but no consistent relationship has been established. In this study, we employed an integrative analysis that combined Mendelian randomization (MR) and bioinformatics analyses to comprehensively explore the association between DR and adiponectin, aiming to provide a unified answer of their relationship. Using the inverse-variance weighted (IVW) method, the odd ratio (OR) of developing DR per 1 mg/dL increment in genetically predicted log-transformed adiponectin concentration was estimated to be 0.949 (*P* = 0.557). Other robust MR methods produced consistent results, confirming the absence of a causal effect of adiponectin on DR. Additionally, the expression levels of the six adiponectin-related genes showed no significant differences among normal controls, individuals with diabetes but without DR, and those with DR Furthermore, the biological pathways enriched by these genes were not strongly relevant to DR. At both the individual gene and pathway levels, there were no overlaps between the adiponectin-related genes and the differentially expressed genes, indicating a lack of association between adiponectin and DR based on gene expression profiles. In summary, the integrative analysis, which combined MR and bioinformatics data mining, yielded compelling evidence supporting the notion that adiponectin is not a risk factor for DR.

## Introduction

Diabetes, a prevalent chronic disease with very high prevalence in both developed and developing countries, imposes a significant global public health burden [1]. Diabetic patients may develop microvascular complications, including retinopathy, nephropathy, and neuropathy, depending on the duration and severity of hyperglycaemia [2]. Diabetic retinopathy (DR) is the leading cause of vision impairment and blindness in adults, characterized by damages to the retinal blood vessels and nerves [3]. More than 60% of diabetic patients may develop DR, which is a major cause for blindness in diabetics aged > 20 [3]. Therefore, it is crucial to gain a comprehensive understanding of the pathophysiological mechanisms underlying the onset and progression of DR to enable early prevention and effective therapeutic interventions.

Adiponectin, an adipocytokine, plays an important role in human metabolism, such as lipid regulation, glucose metabolism, and thus mediates the body’s response to insulin [4]. Numerous epidemiological studies have explored the association between adiponectin and DR, but inconsistent conclusions have been reached. Some studies have reported higher serum adiponectin levels in DR patients compared to non-DR patients [5–8]. For instance, Yang et al [5] conducted multivariate logistic analyses demonstrating strong correlations between serum and aqueous adiponectin levels and the risk of DR. Conversely, a few observational studies, such as [9], have reported lower levels of serum adiponectin in DR patients compared to non-DR patients. Moreover, a meta-analysis of 19 relevant observational studies involving 1545 DR patients and 1502 controls [10] also identified a negative correlation between adiponectin levels and the risk of DR.

The examination of transcriptomic data, such as mRNA molecules, has the potential to elucidate the involvement of specific genes in complex diseases [11], such as heart disease [12], lung cancer [13,14], and diabetes [15]. To date, numerous studies have been conducted to identify relevant gene signatures associated with DR, utilizing transcriptomic data and bioinformatics methods [16–19]. For instance, Gu et al. [16] employed several conventional bioinformatics approaches on gene expression data including differentially expressed gene analysis, pathway enrichment analysis, to identify angiogenesis-related genes and pathways in early DR. Notably, the predominant study design used in transcriptomic experiments, such as microarray or RNA-Seq studies, is cross-sectional, allowing only the exploration of potential associations between genes and the outcome of interest can be examined, without establishing causality.

On the other hand, Mendelian randomization (MR) studies have emerged as a new epidemiological strategy to ascertain causal relationships between traits and the disease under investigation [20]. MR leverages the random allocation of genetic variants, often single nucleotide polymorphisms (SNPs) as instrumental variables (IVs). As environmental traits cannot modify DNA, reverse causality and confounding effects are minimized. Moreover, due to the static nature of genetic variants, the MR approach mitigates measurement errors. It is anticipated that combining MR analysis with transcriptomic data analysis can offer further insight into the onset and progression of specific diseases.

However, studies that integrate gene expression data analysis and MR analysis to examine the relationship between the exposures and the outcome of interest are limited. In this study, we aim to explore the potential synergistic value of such integration (in other words, to investigate whether they can complement to each other to provide a more comprehensive evaluation). Therefore, we performed both MR analysis and transcriptomic data analysis to examine the relationship between adiponectin and DR from diverse perspectives.

## Materials and methods

### MR analysis

To investigate the potential causal role of adiponectin in the development and progression of DR, a two-sample MR analysis was conducted using the online platform MR-Base (https://app.mrbase.org) [21]. Adiponectin abundance was considered as the exposure variable, while DR was the outcome of interest. Multiple SNPs associated with adiponectin at a genome-wide significance level (*P* < 5 × 10^−8^), with low linkage disequilibrium (*R*^*2*^ <0.001) were selected from the AIPOGen consortium. This consortium comprised individuals from diverse ethnic backgrounds, including white Europeans (n  =  29347), African Americans (n  =  4,232), and East Asians (n  =  1,776) [22]. Summary data from the FinnGen consortium, which aims to collect and analyse genome and health data from 500,000 Finnish Biobank participants, were utilized for the diabetic retinopathy genome-wide association study (GWAS) study. The FinnGen consortium releases summary data twice a year, according to their agreement. The data used in the MR-Base platform, which included 14,584 cases and 176,010 controls of European ancestry, were recently released on 11 May 2021 (https://www.finngen.fi/en).

The inverse-variance weighted (IVW) method was employed to estimate the effect size, representing the impact of a one-unit increase in the natural log-transformed adiponectin increase on the susceptibility to diabetic retinopathy. This estimation allows for the evaluation of the causal relationship between adiponectin and DR. The MR analysis faced the challenge of addressing pleiotropic effects, as horizontal pleiotropy can lead to erroneous causal effect assignment. To comprehensively assess the presence of horizontal pleiotropy and then minimize its impact, more pleiotropy-robust methods such as MR-Egger regression [23], the Weighted Median Estimator [24], and the Weighted Mode method [25] were employed. The MR-Base platform also provided tests for assessing SNP heterogeneity, including funnel plots, Cochrane’s Q tests, and leave-one-out sensitivity analyses. It is worth noting that leave-one-out sensitivity analysis can also identify outliers (SNPs with extreme effects deviating from others) and potential horizontal pleiotropy.

The MR study adhered to the STrengthening the Reporting of Observational studies in Epidemiology-MR (STROBE-MR) checklist [26]. Additionally, a post-hoc statistical power calculation for the MR analysis was conducted using the mRnd online calculator (https://cnsgenomics.com/shiny/mRnd) [27] to determine whether the present MR study has sufficient power.

## Transcriptomic data analysis

### Experimental data

The raw data for the DR cohort were obtained from the Gene Expression Omnibus (GEO: https://www.ncbi.nlm.nih.gov/geo/) repository (accession number: GSE160306). This RNA-seq experiment [19] consisted of 76 subjects categorized into three groups: 20 healthy controls, 17 individuals with diabetes but without DR, and 39 DR patients with varying degrees of severity. The expression levels, represented as normalized log2 Counts Per Million (CPM) mRNA values, were determined using the R limma voom function [28].

## Statistical methods

Moderated t-tests were carried out to identify differentially expressed genes (DEGs) using R limma package; a Venn diagram was employed to investigate the interaction of identified DEGs across three different comparisons. Similarly, another Venn diagram was utilized to explore the overlaps between the identified DEGs and adiponectin-associated genes obtained from the dbSNP database [29]. The identified genes were visually represented, and patterns within their expression profiles were examined using a t-distributed stochastic neighbour embedding (tSNE) plot. tSNE is a widely used non-linear dimensionality reduction method, particularly suitable for visualizing high-dimensional data in two or three dimensions.

Separate protein-to-protein interaction (PPI) networks were constructed for the identified DEGs and adiponectin-related genes. Subsequently, Gene Ontology (GO) [30] functional analysis and Kyoto Encyclopedia of Genes and Genomes (KEGG) [31] pathway enrichment analysis were performed using the Search for the Retrieval of Interacting Genes/Proteins (STRING) software [32]. The biological relevance of DEGs, adiponectin-related genes, and their enriched pathways was further investigated using the GeneCards knowledgebase [33] and PubMed mining.

In summary, the proposed procedure firstly applied Kruskal-Wallis nonparametric tests to assess whether the expression values of adiponectin-related genes significantly differed across the three groups. Pathway enrichment analyses were then conducted for the adiponectin-related genes to determine potential associations between these genes and DR at the pathway level. Next, DEGs were identified for the three comparisons, and their intersections with the adiponectin-related genes were visualized using Venn-diagrams. Finally, pathway enrichment analyses were performed for the DEGs to examine if the resulting enriched pathways overlapped with those enriched by the adiponectin-related genes, thereby evaluating the presence of a pathway-level connection between adiponectin and DR.

All bioinformatics analysis was carried out in the R software, version 4.1.0 (http://www.r-project.org/).

## Results

### Results of MR analysis

A power calculation was performed before the formal MR analysis to determine whether the current MR study was adequately powered. A sample size of 190,594 (the sample size of the FinnGen study) had a power of 0.07–0.29 to detect a causal risk of 0.9 to develop DR when the genetically predicted adiponectin level increases by one unit at a significance level of 0.05 (the related genetic variants explained away 0.11% − 1.47% of the variance in the level of adiponectin). Overall, all SNPs together explained away 5.54% of the variance in the level of adiponectin (assuming these SNPs are independent from each other), thus the MR study had a power of 0.78 to detect a causal risk of 0.9 to develop DR when the genetically predicted adiponectin level increases by one unit at a significance level of 0.05.

**Figure 1.**
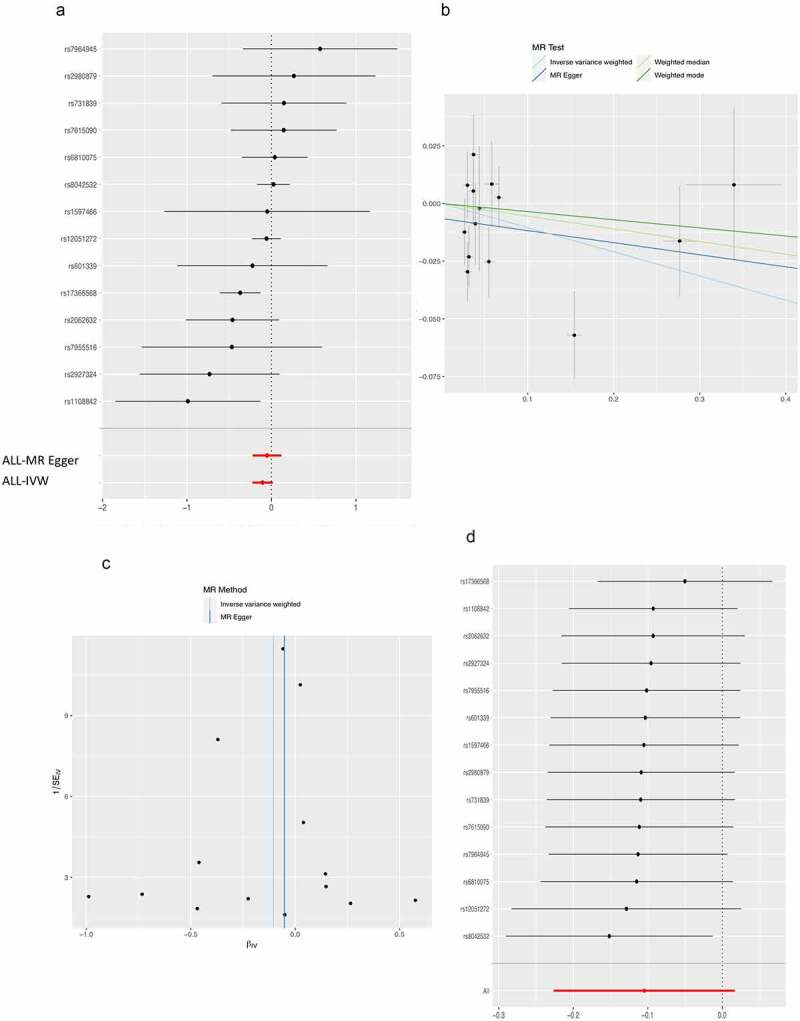
Results of the Mendelian randomization analysis investigating the causal effect of adiponectin on diabetic retinopathy. a) Forest plot displaying the effect estimates. b) Comparison of the four MR analysis methods employed. c) Funnel plot assessing directional horizontal pleiotropy. d) Leave-one-out analyses detecting outliers.

The MR analysis including 14 SNPs (the details on these 14 SNPs were provided in Supplementary Table S1) was then performed using the MR-base online app. No pleiotropic effects of 14 adiponectin-associated SNPs (the intercept of MR-Egger regression was −0.007, *P* = 0.398) were found. These SNPs exhibited high homogeneity according to Cochran’s Q statistics (Q value = 18.15, *P* = 0.111 for the MR Egger method, and Q value = 19.31, *P* = 0.114 for the IVW method) and the funnel plot. Based on 14 adiponectin-associated SNPs, no causal association between adiponectin and DR was found, and the null results showed concordance across all MR analysis methods considered. Specifically, the risk of developing DR when the genetically predicted log-transformed adiponectin level increased 1 ln (mg/dL) was reduced to 0.949 (*P* = 0.557) by the IVW method. The MR-Egger method estimated the odds ratio (OR) to be 0.947 (*P* = 0.432) the weighted median method to be 0.901 (*P* = 0.091) and the weighted mode method to be 0.965 (*P* = 0.596) Figures 1(a,b) Leave-one-out sensitivity analysis and a funnel plot reveal no SNPs significantly influenced the causal association of adiponectin and DR Figures 1(c, d)

Due to the relatively small sample size of the GWAS study is relatively small, a sensitivity analysis was performed by setting the genetic significance level at 10^−5^ instead of 5.0 × 10^−8^. Consequently, 34 SNPs were identified as IVs using the 10^−5^ threshold. The results of the MR analysis using these 34 SNPs as IVs are basically similar to the results obtained with 14 SNPs, with one exception. The IVW method indicated a negative causal relationship between adiponectin and DR (OR = 0.905, *P* = 0.037). The MR-Egger method estimated an OR of 0.952 (*P* = 0.447), the weighted median method estimated an OR of 0.949 (*P* = 0.440), and the weighted mode method estimated an OR of 0.972 (*P* = 0.645). Additionally, these SNPs exhibited high homogeneity (Cochran’s Q value = 36.52, *P* = 0.267 for the MR Egger method, and Cochran’s Q value = 38.24, *P* = 0.244 for the IVW method).

Considering that the exposure GWAS study included a diverse population with three different ethnicities, another sensitivity analysis was conducted, focusing only on individuals of European descent in the exposure GWAS study. Notably, the null causal results remained consistent in this analysis.

## Results of gene expression analysis

**Figure 2.**
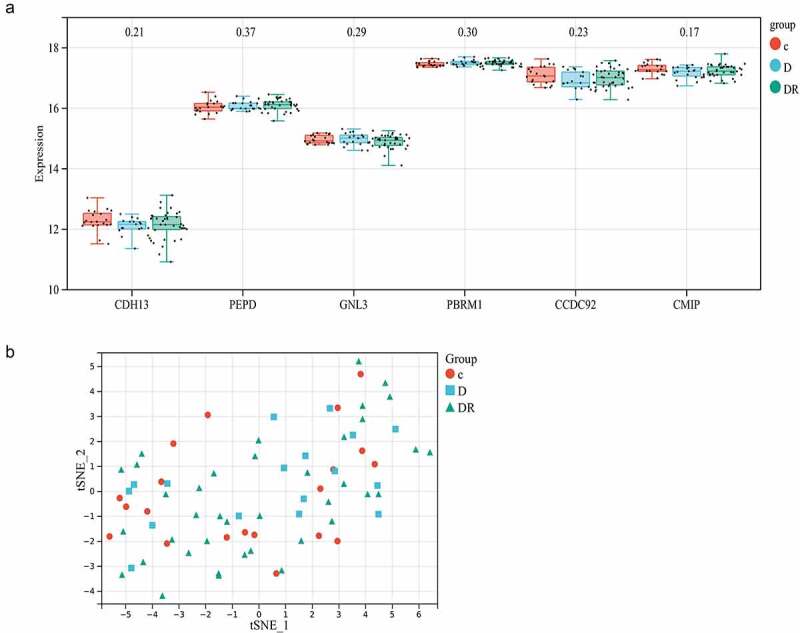
Results of the transcriptomic data analysis. a) Boxplots representing the expression values of adiponectin-related genes, accompanied by the corresponding p-values obtained from non-parametric test p-values. The boxplots illustrate gene expression values along the y-axis, with the gene symbols of the six adiponectin-related genes listed on the x-axis. These boxplots demonstrate that none of the adiponectin-related genes displayed significant differential expression among the three groups. Here, p-value indicate the corresponding p-values of Kruskal-Wallis non-parametric tests conducted on each specific genes. b) 2D tSNE scatterplot showing these 6 genes had no discriminative ability to distinguish the three groups. To visualize the segmentation results obtained from the 6 genes, a tSNE scatterplot was generated. The x-axis and y-axis represent the first two tSNE components. The scatterplot clearly demonstrates that the samples from the three different groups are intermingled, indicating that these 6 genes lack discriminative ability. C: Normal control; D: diabetic group without retinopathy; DR: diabetic retinopathy group.

The genes associated with the 14 adiponectin-related SNPs were obtained from the dbSNP database [29]. These genes included ADIPOQ (rs17366568), ADIPOQ-AS1 (rs17366568), GNL3 (rs1108842), PBRM1 (rs1108842), KNG1 (rs2062632), CMIP (rs2927324), LOC107986141 (rs1597466), PEPD (rs731839), CCDC92 (rs7964945) and CDH13 (rs12051272). It is worth mentioning that 4 of these genes were not annotated on the RNA-Seq platform. By performing Kruskal-Wallis non-parametric tests, it is observed that none of remaining 6 genes exhibited significant differential expression across the three groups: normal controls, individuals with diabetes but without DR, and DR patients. This observation held even without adjusting for multiple testing (Figure 2a). Additionally, the tSNE scatterplot demonstrated that these 6 genes did not possess discriminative values to distinguish between the control, diabetic without DR, and DR groups (Figure 2b).

GO and KEGG pathway enrichment analysis revealed that these genes were significantly enriched in two GO biological process (BP) terms (Nucleosome disassembly and Regulation of fatty acid biosynthetic process), six GO cellular component (CC) terms (Preribosome, PeBow complex, SWI/SNF superfamily-type complex, npBAF complex, nBAF complex and SWI/SNF complex), two GO molecular function (MF) terms (Adiponectin binding and Adipokinetic hormone receptor activity), and six KEGG pathways (Hepatocellular carcinoma, Adipocytokine signalling pathway, Longevity regulating pathway, Complement and coagulation cascades, AMPK signalling pathway, and Non-alcoholic fatty liver disease).

In the literature, no studies showed that these pathways are related to DR with few exceptions, taking AMPK signalling pathway [34], longevity regulating pathway [35], and complement and coagulation cascades [36] that had been demonstrated to correlate with the occurrence, progression and therapeutic intervention of DR as examples. Although the identified adiponectin genes are involved in those DR-relevant pathways, other genes such as SIRT1 [37] and MTOR [34] should play key roles in the development of DR. Conversely, ample evidences in the literature [38–41] supported the biological significance of these enriched pathways in diabetes. Therefore, we cannot rule out the possibility that the insufficient evidence of these enriched pathways related to DR is due to the shortage of such work. Further research is needed to elucidate the role of the identified genes and pathways in development and progression of DR.

**Figure 3.**
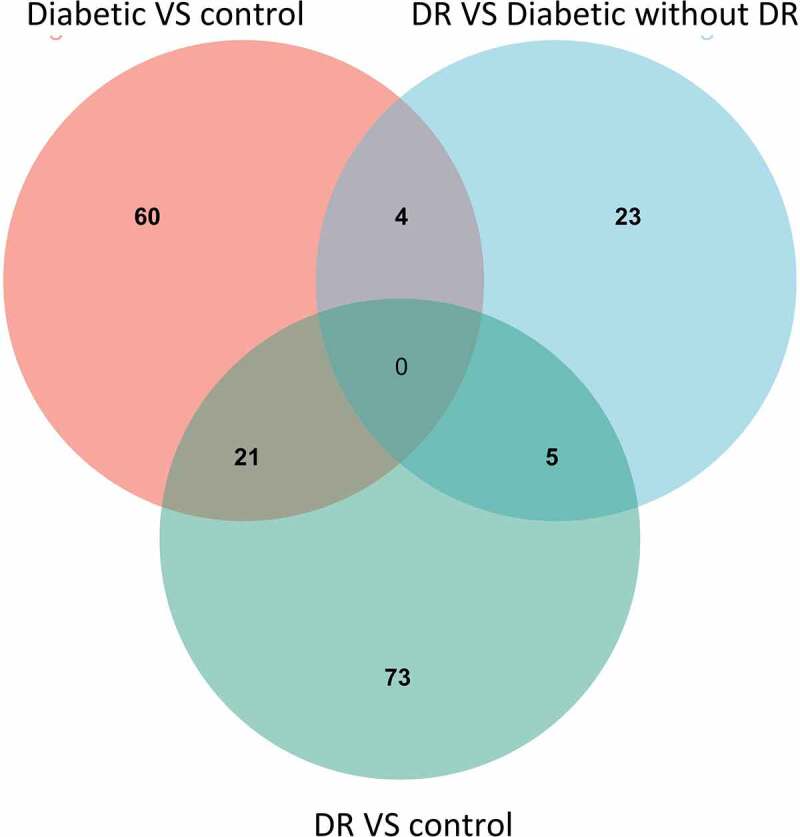
Venn diagrams showing how the identified differentially expressed genes (DEGs) overlapped across the three comparisons. This Venn diagram demonstrates the absence of shared genes between these three gene sets.

The R limma package was employed to identify differentially expressed genes (DEGs). The cut-off values of fold change (FC) and p-value were set at 1.1 and 0.01, respectively. The comparison between the diabetic group without DR and the control group revealed 40 up-regulated and 45 down-regulated genes. There were 64 overexpressed and 35 for under-expressed genes found in the comparison between the diabetic group with DR and the control group, whereas 20 were up-regulated and 12 were down-regulated in the comparison between the diabetic with DR group versus without DR group. Venn diagrams (Figure 3) demonstrated that there were no genes common across the three lists of DEGs, which comprised a total of 186 unique genes. Additionally, no gene was shared between DEGs and the previously mentioned six adiponectin-related genes. It is noteworthy that the crude p-values were utilized in these analyses due to the limited number or absence of DEGs when adjusted p-values were applied. Interestingly, based on the shared four DEGs (S100B, CD14, TLX2 and ILI2-AS1) between diabetes without DR versus controls and DR versus diabetes without DR, the change trajectories observed were non-monotonic, with neither a strictly increasing nor decreasing pattern.

Table 1 displays the enriched GO terms and KEGG pathways using the String software. No overlapping pathways were observed between the lists of DEGs and the adiponectin-related genes. The collective findings from the bioinformatics analyses, considering both individual genes and associated pathways, suggest that the adiponectin level was unlikely to relate to the risk of DR.

**Table 1.** Enriched GO terms and KEGG pathways by the identified differentially expressed genes.

Pathway ID	Description	# of gene	Background	FDR
GO terms				
GO:0000781	Chromosome telomeric region	9	134	0.0059
GO:0000784	Nuclear chromosome telomeric region	8	102	0.0059
GO:0003688	DNA replication origin binding	5	23	0.0108
GO:0043394	Proteoglycan binding	5	37	0.0425
GO:0006270	DNA replication initiation	6	32	0.0092
GO:0030334	Regulation of cell migration	23	865	0.0102
GO:0001568	Blood vessel development	16	500	0.0192
GO:0045807	Positive regulation of endocytosis	8	103	0.0192
GO:0051270	Regulation of cellular component movement	24	1009	0.0192
GO:0006261	DNA-dependent DNA replication	8	119	0.0195
GO:0022603	Regulation of anatomical structure morphogenesis	24	1095	0.0272
GO:0048844	Artery morphogenesis	6	61	0.0272
GO:0050793	Regulation of developmental process	42	2648	0.043
GO:1901342	Regulation of vasculature development	12	336	0.043
GO:0030335	Positive regulation of cell migration	15	522	0.0461
GO:0033003	Regulation of mast cell activation	5	43	0.0461
KEGG				
hsa04110	Cell cycle	7	120	0.0339

Moreover, an integration of the DEGs and adiponectin-related genes (a total of 196 genes, with 174 genes annotated in the String software) was conducted. GO and KEGG pathway enrichment analysis of this gene set demonstrated significant enrichment in 27 GO terms and 2 KEGG pathways. One KEGG pathway overlapped with the enriched KEGG pathways identified by the adiponectin-related genes, while 15 GO terms and 1 KEGG pathway overlapped with the enriched pathways identified by the DEGs. Among the unique 12 GO terms identified by the union of these two gene lists, their corresponding false discovery rates (FDRs) ranged from 0.02 to 0.05, and none of them have been previously reported to be associated with DR. Notably, none of the GO terms enriched by the adiponectin-related genes were included in this list, which is not surprising given that the proportion of adiponectin-related genes in the union list is relatively small compared to the DEGs.

Furthermore, to mitigate potential biases arising from the different categories used in the MR analysis and transcriptomics analysis, another sensitivity analysis was performed. In this analysis, the normal controls and individuals with diabetes but without DR were combined into a larger group, and the limma package was employed to identify DEGs between DR and this newly formed group (controls + diabetes without DR). To be consistent, the same cut-off values for fold change (FC) and p-value as the previous DEG analyses (1.1 and 0.01, respectively) were used. This analysis identified 70 up-regulated genes and 204 down-regulated genes. Once again, there were no overlapped genes between these 274 DEGs and the adiponectin-related genes.

## Conclusions and discussion

As the first study that integrated MR and bioinformatics analysis to investigate the relationship (which is not limited to the association but extended to causality) between adiponectin and DR, the current study in line with a previous MR study [42], strengthen the view that adiponectin is not a risk factor of DR. The association observed in previous epidemiologic studies [5–8] may be attributed to reverse causality or unaccounted confounding factors. Indeed, demographic characteristics, including age, BMI range, ethnicity, and types of diabetes were found to be heterogeneous in the aforementioned observational studies, explaining some of contradictory findings. More importantly, a small or at most moderate sample size is a common issue in those observational studies.

The current study has its own strengths. The MR design alleviates the influence of confounding factors and reverse causality, to which the targeted bioinformatics analyses were added. Such integrative analyses utilized multiple omics data while with a particular focus on adiponectin. To our knowledge, the present study is among the first efforts performing this type of analysis. The results of MR analysis and bioinformatics analysis were in concordance with each other, making the final conclusion more robust and persuasive. Regarding the bioinformatics analyses, none of adiponectin-related genes were identified as DEGs, thus these SNPs may not act as eQTL(expression quantitative trait loci) for DR. Besides the conventional differentially expressed gene analysis, the possible underlying mechanisms and functions adiponectin-related genes may play were also explored via pathway enrichment analysis. The association between adiponectin-related genes and DR was comprehensively investigated at the levels of both pathway and individual gene.

Certainly, the study also has its own limitations. First, due to ethnic differences in the populations of exposure and outcome GWAS studies, residual confounding from population stratification cannot be completely excluded in the two-sample MR setting. However, a sensitivity analysis using only white Europeans (*n* = 29,347) from the exposure GWAS study was conducted, and the results were consistent with the finding from the entire APIOGen consortium. Additionally, the conventional MR settings in this study were unable to detect a non-linear causal relationship between exposure and outcome. No trend tests were performed on the expression values across different stages of DR: neither linear nor non-linear trends were explored. Second, according to the sample size calculations conducted by us, the sample size of the GWAS study under consideration may be insufficient to detect a subtle causal effect, as the sample size of the RNA-Seq experiment considered in this study was small, resulting in the identification of only few DEGs. Third, the tissues studied for both gene expression profiles and MR analysis differed, namely, retina versus blood, let alone the gene expression level is not equal to (may not even be significantly related to) the abundant level of a marker/protein in the same issue. Finally, the Finnish population differs from conventional European ancestry regarding linkage disequilibrium (LD) structure and allele frequency, which may introduce more bias into MR analysis.

In conclusion, this study found no evidence to support a link between adiponectin levels and the development of DR. Therefore, adiponectin can not serve as a biomarker or potential therapeutic target for DR.

## Supplementary Material

Supplemental Material

## Data Availability

Data of microarray experiment used were downloaded from the Gene Expression Omnibus (GEO: https://www.ncbi.nlm.nih.gov/geo/) repository.
